# Society Proceedings

**Published:** 1898-07

**Authors:** 


### SOCIETY PROCEEDINGS.

### PENNSYLVANIA STATE VETERINARY MEDICAL ASSOCIATION.

*Officers and Committees*, 1898–1899.

President, George B. Jobson, Franklin, Pa.; First Vice-President, J. Curtis Michener, Colmar, Pa.; Second Vice-President, J. F. Butterfield, South Montrose, Pa.; Third Vice-President, F. F. Hoffman, Brookville, Pa.; Treasurer, Francis Bridge, Philadelphia, Pa.; Recording Secretary, Jacob Helmer, Scranton, Pa.; Corresponding Secretary, W. L. Rhoads, Lansdowne, Pa.

Legislative Committee: Chairman, W. Horace Hoskins, 3452 Ludlow Street, Philadelphia, Pa.; B. F. Bachman, Strasburg, Pa.; W. H. Ridge, Trevose Pa.; J. W. Sallade, Pottsville, Pa.; Leonard Pearson, 3608 Pine Street, Philadelphia, Pa.; Thomas B. Rayner, Highland Avenue, Chestnut Hill, Philadelphia, Pa.; J. C. McNeil, 26 Fourth Street, Pittsburg, Pa.

Committee on Intelligence and Education: Chairman, H. B. Felton, Olney, Pa.; J. F. Butterfield, South Montrose, Pa.; W. S. Kooker, 1116 Wallace Street, Philadelphia, Pa.; C. C. McLean, Meadville, Pa.; James A. Waugh, 813 Forbes Street, Pittsburg, Pa.; C. R. Good, Lock Haven, Pa.; R. J. Mumma, Hanover, Pa.

Committee on Sanitary Science and Police: Chairman, Leonard Pearson, 3608 Pine Street, Philadelphia, Pa.; Otto G. Noack, Reading, Pa.; Clarence J. Marshall, 2022 Pine Street, Philadelphia, Pa.; J. Stewart Lacock, 38 Diamond Street, Allegheny City, Pa.; E. M. Ranck, 427 N. Forty-first Street, Philadelphia, Pa.; R. C. Rice, Towanda, Pa.; J. B. Irons, Erie, Pa.

Trustees: James B. Rayner, West Chester, Pa.; W. Horace Hoskins, 3452 Ludlow Street, Philadelphia, Pa.; W. H. Ridge, Trevose, Pa.; J. C. McNeil, 26 Fourth Street, Pittsburg, Pa.; James W. Sallade, Pottsville, Pa.

### VETERINARY MEDICAL ASSOCIATION OF NEW YORK COUNTY.

In the absence of the President and Vice-President, the regular monthly meeting was called to order June 1, 1898, at 8.45 P.M. by the Secretary. On motion, the Chairman of the Board of Censors was voted to the chair. The following members responded to roll-call: Drs. Amling, C. C. Cattanach, J. S. Cattanach, Ellis, Gill, Lamkin, Lellman, Machan, MacKellar, O'Shea, and Ryder. The minutes of the previous meeting were read and approved.

Dr. Gill, Chairman of the Board of Censors, reported as follows: Whereas, at the March meeting Dr. Huidekoper, on the impulse of the moment, unintentionally slighted Dr. Bell, by failing to recognize Dr. Ackerman in the debate, as per programme arranged by Dr. Bell as Chairman of the Ways and Means Committee, and Dr. Huidekoper, having recognized his error, and having made an apology to Dr. Bell, it is resolved that the Secretary be instructed to ask Dr. Bell to withdraw his resignation as Chairman of the Ways and Means Committee. Moved and seconded that the report be accepted; carried.

Dr. Lellman read a paper entitled, "Multiple Sclerosis of the Brain and Spinal Cord of a Dog."[1] Moved and seconded that a vote of thanks be extended to the essayist; carried.

Dr. Ryder, Chairman pro tem. of the Ways and Means Committee, stated that he thought it was the function of this committee to attend to any such arrangements as were to be made for the reception of Veterinary Medical Society of the State in September.

Dr. O'Shea, for the Judiciary Committee, reported that this committee had received several reports of illegal practitioners, but so far were unable to secure specific evidence against the parties accused, and, until they could do so, the committee deemed it unwise to take any action in the matter whereby the Association would be made liable. Moved and seconded that the report be accepted; carried.

A communication to the Association was read by the Secretary, as follows:

DR. R. W. ELLIS, Secretary Veterinary Medical Association of New York County, New York City,

SIR: I have the honor to submit my resignation to the Veterinary Medical Association of New York County.

Will you please present to the members my thanks for their courtesy and my hopes for their well-being and the success of the Association.

Very faithfully yours,
RUSH S. HUIDEKOPER,
Lieutenant-Colonel and Chief Surgeon,
Care of War Department, Washington, D. C.

Discussion. Moved and seconded that the matter be referred to the Board of Censors; carried.

Moved and seconded that a recess of ten minutes be taken while the board could take action on Dr. Huidekoper's resignation; carried.

The Board of Censors subsequently offered the following report:

We recommend that the resignation of Dr. Huidekoper be accepted, and that the Secretary be authorized to notify Dr. Huidekoper of the same, expressing the Association's well wishes for him in his new field of labor.

Moved and seconded that the meeting adjourn; carried.

ROBERT W. ELLIS, D.V.S.,
Secretary.

### MANITOBA VETERINARY ASSOCIATION.

THE annual meeting of the Association was held at Winnipeg, February 15, 1898. Drs. Hinman, F. Torrance, Dunbar, Swinnerton, Thompson, Little, Spiers, Hilliard, Hilton, Williamson, Robinson, and Nagle were present.

Several amendments to the by-laws were considered and read.

Examination fee of candidates desiring to practise in the province was raised to twenty-five dollars, which, in the event of failure to pass, would entitle them to a second examination within six months.

The receipts of the Association showed $535.59, and the expenditures $213.36, leaving on hand in the treasury $322.23.

The province has forty-nine qualified practitioners, and two have not completed qualifications.

[1] See page 466.

The council of the Association elected for the year 1898 are: Drs. Dunbar, Torrance, Young, Little, Spiers, Swinnerton, and Hilton. The council selected Dr. Little for President; Dr. Spiers, Vice-President; Dr. Dunbar, Secretary-Register; Drs. Torrance, Dunbar, and Little, Examiners of the Association ; Drs. Williamson and Robinson, Auditors. The annual fees were fixed at two dollars.

Dr. Hilton presented a paper on " The Progress of Veterinary Science." It was discussed and commended by members Thompson, Dunbar, Torrance, and Spiers.

Dr. Hilliard presented a paper on " Influenza." The paper was commended for its concise presentation of the subject, and discussed by Drs. Hinman, Torrance, and Dunbar.

The semiannual meeting will be held in Winnipeg. The subjects of ovariotomy, dehorning, and secondary hemorrhage were discussed and reported upon.

The proceedings were ordered to be printed for distribution among the members. Infractions of the laws governing the practice of veterinary science in Manitoba were reported upon, discussed, and referred to the council.

*Some Features of the Manitoba Veterinary Association.*

The Manitoba Veterinary Association is an incorporated body.

It conducts all examinations of those desiring to practise in the province. The fees for the same go into the general treasury of the Association. An average of 75 per cent. must be obtained to pass.

All prosecutions and investigations are conducted under the auspices of the Association, and placed in charge of the council.

Only qualified members are eligible to membership in the Association.

Members of the Association are protected in litigation by the council. Unprofessional conduct of any member is punished by debarment from the privileges of prosecution, defense, and other safeguards of the society.

The various acts and amendments relative to the Association were consolidated on March 19, 1896. After 1898 all applicants for membership and practice in the province must be graduates of colleges with curriculums of three years of six months each.

Only those provided for under this act shall have the right to collect fees for professional services, cost of medicines, or medical appliances supplied in a professional capacity.

No district veterinarian or veterinary surgeon in any branch of the public service of the province can be appointed unless registered as a member of the Association, and those duly registered shall be entitled to professional fees for giving evidence in such cases as relate to the veterinary profession. Castration and spaying are exempted from the provisions of the act.

Penalties are provided for violations, and methods of procedure are provided.

The Association is further privileged to hold real-estate not in excess of five thousand dollars, and may use this as it sees proper; may erect buildings for its work, and for lectures and teachers in connection therewith, and for a library and other purposes proper for the objects of the Association.

### VETERINARY MEDICAL SOCIETY OF THE ONTARIO VETERINARY COLLEGE.

THE last regular weekly meeting of the Society for the present college year was held March 11th. During the college session one hundred and fifty-six papers were read on various subjects of interest to the students. The discussions were exceedingly interesting and instructive, thus impressing many valuable points upon the minds of the students. The following is a list of the papers read during the past month:

Essays: "Rinderpest," T. I. Fletcher; "Gid," B. W. Groff; "Hernia," W. L. Adams; "Actinomycosis," E. T. Cunningham; "Aloin," T. Lambrechts; "Incompatibility," Hamlet Moore; "Rabies," H. W. Stedman; "Heredity," G. P. Hayter; "Cocaine," G. K. Cranston; "Anthrax," C. Owens; "Azoturia," A. D. McLachlin; "Barium Chloride," T. Rowland; "Shoeing," J. A. McDonald; "Nux Vomica," W. E. Fairbanks; "Lithotomy," G. W. Higginson; "Ophthalmia," F. M. Hayward; "Chlorodyne," J. P. Howland; "Ovasectomy," E. B. Truitt; "Creolin," J. Short; "To Class of '98," W. H. Corey.

Communications: "Punctured Wounds," A. H. Krull; "Acute Indigestion," S. Caldbick; "Fistula of Steno's Duct," D. S. Jones; "Gunshot Wound," J. W. Rutledge; "Diarrhœa," A. P. Lubach; "Wisdom of Examining Foot," J. S. McIntyre; "Œsophagotomy," J. P. Howland; "Tympanitis," E. B. Truitt; "Pectoral Injury," J. Dixon; "Strychnine Poisoning in Dog," C. Owens; "Spasm of Diaphragm," A. G. Van Tine.

C. W. FISHER,
Secretary.

*Resolutions of the Ontario Veterinary Medical Association.*

The Ontario Veterinary Medical Association has adopted the following resolutions: 1. That the tuberculin-test needs to be safeguarded by certain precautions not possible to those without professional experience. 2. That it is not in the interests of the farmers of this province to have any person authorized to instruct them in the use of tuberculin as a test for tuberculosis, as it is liable to create unnecessary alarm concerning the health of the cattle of Ontario. 3. That a continuation of the present methods will bring the tuberculin-test into disrepute, for the following reasons: That animals destined for shipment to the United States may be injected with tuberculin by the owners, and the subsequent test of the Dominion Veterinary Inspector rendered both useless and deceptive, paving the way for fraud and consequently injuring the livestock trade for this country.

